# Early detection of knee osteoarthritis – The role of a composite disease activity metric: Data from the Osteoarthritis Initiative

**DOI:** 10.1016/j.ocarto.2026.100811

**Published:** 2026-05-05

**Authors:** Julieann C. Patarini, Timothy E. McAlindon, Jonggyu Baek, Emily Kirillov, Nhung Vo, Michael J. Richard, Ming Zhang, Matthew S. Harkey, Grace H. Lo, Shao-Hsien Liu, Kate Lapane, Charles B. Eaton, Jamie MacKay, Jeffrey B. Driban

**Affiliations:** a Department of Population and Quantitative Health Sciences, University of Massachusetts Chan Medical School, Worcester, MA, USA; b Division of Rheumatology, University of Massachusetts Chan Medical School, Worcester, MA, USA; c Division of Rheumatology, Allergy, & Immunology, Tufts Medical Center, Boston, MA, USA; d Department of Computer Science, Boston University, Boston, MA, USA; e Department of Kinesiology, Michigan State University, East Lansing, MI, USA; f Department of Medicine, Baylor College of Medicine, Houston, TX, USA; g Medical Care Line and Research Care Line, Houston VA HSR&D Center for Innovations in Quality, Effectiveness and Safety, Michael E. DeBakey Medical Center, Houston, TX, USA; h Center for Primary Care and Prevention, Kent Hospital, Pawtucket, RI, USA; i Brown University School of Public Health, Providence, RI, USA; j Department of Radiology, University of Cambridge, Cambridge, UK; k Norwich Medical School, University of East Anglia, Norwich, UK

**Keywords:** Bone marrow lesions, Effusion synovitis, Knee, Magnetic resonance imaging

## Abstract

**Objective:**

To evaluate whether dynamic disease processes in the knee (effusion-synovitis volume and bone marrow lesions; BML), summarized by a validated efficient continuous composite metric, are prognostic of incident symptomatic knee OA (sxKOA) over the subsequent three years.

**Methods:**

We analyzed knee magnetic resonance (MR) images from a sample of Osteoarthritis Initiative participants without sxKOA using a previously validated osteoarthritis disease activity metric that provides a normalized continuous score reflecting effusion-synovitis and BML volumes throughout the knee. The outcome was incident sxKOA during the subsequent three years, defined by the report of frequent knee pain and presence of radiographic OA (Kellgren-Lawrence grade ≥2). We used a modified Poisson model to assess the association between disease activity (continuous measures; relative risk [RR] per 1 unit of measure) and incident sxKOA, adjusting for gender, age, and body mass index.

**Results:**

Among 913 knees (572 participants; 56% female; mean (SD): 61 (9) years of age), disease activity ranged from −3.83 to 24.79 (higher values = greater pathology). Knees with greater disease activity (RR = 1.08 per 1 unit of disease activity [95% confidence interval: 1.04–1.11]) had a greater risk of incident sxKOA.

**Conclusion:**

Effusion-synovitis and BML volumes – represented by a validated composite score – predict the future incidence of sxKOA. This finding indicates that dynamic pathophysiologic processes are operative in knees before sxKOA becomes overt. This scenario could represent early knee OA. Moreover, the pathophysiological processes represented by effusion-synovitis and BMLs on MR images, much of which are probably inflammatory, could suggest therapeutic targets for preventive intervention.

## 1 Introduction

Osteoarthritis (OA) is a painful disorder that affects over 500 million people globally [1]. Despite a promising therapeutic development pipeline, no therapies have been proven to reduce OA disease progression [2,3]. Much of the focus of clinical research in OA, until recent years, has focused on those with established symptomatic OA. Given the challenge to find a disease-modifying therapy for knee OA, many have postulated that a substantial barrier has been the focus on disease too late in its natural history to alter the disease course [[4], [5], [6], [7], [8], [9], [10], [11]]. Thus, there is a growing focus on methods to identify knees at earlier stages of OA when the therapeutic opportunity is plausibly greatest [[4], [5], [6], [7], [8], [9], [10], [11]]. Identifying clinically meaningful disease processes during the early stages of OA could also reveal therapeutic targets and promising biomarkers to monitor therapeutic effects.

During the last 3 decades, evidence has revealed that bone marrow lesions (BMLs) and effusion-synovitis are frequently detected from early to late stages of knee OA [[12], [13], [14], [15], [16]] and associated with knee symptoms [13,17]. We recently demonstrated that BMLs and effusion-synovitis may reflect a common construct of dynamic disease processes in OA [18]. This construct can be quantified with a magnetic resonance (MR) imaging-based composite biomarker – the disease activity metric – that combines effusion-synovitis and six regional BML volumes [18]. Among knees with OA, changes in the disease activity metric relate to changes in knee pain severity [18]. Understanding the role of this construct of dynamic disease processes in early knee OA could offer vital insights into the pathologic processes that are prognostic of future OA onset, informing therapeutic targets and biomarker development.

Thus, this study aimed to test whether dynamic disease processes (effusion-synovitis volume and BMLs), summarized by the disease activity metric, are prognostic of incident symptomatic knee OA (sxKOA) over the subsequent three years. We hypothesized that the disease activity metric is prognostic of incident sxKOA, which will 1) characterize pathological processes involved in early OA, 2) inform therapeutic targets, and 3) be a valuable biomarker.

## 2 Materials and methods

### 2.1 Study design

To determine the association between the disease activity metric and incident symptomatic knee OA, we used a nested longitudinal cohort of knees without sxKOA from the Osteoarthritis Initiative (OAI) to conduct knee-based analyses (Supplemental Fig. 1). Symptomatic knee OA was operationally defined as a knee with radiographic OA (Kellgren-Lawrence grade ≥2) and self-reported “pain, aching, or stiffness in or around your right knee on most days for at least one month” during the prior 12 months. Incident sxKOA was a knee without symptomatic OA at the 12-month OAI visit but subsequently met the criteria for sxKOA at the 24-, 36-, or 48-month visit.

### 2.2 OAI and source dataset

The OAI is a multicenter cohort study of individuals in the United States with or at risk for sxKOA. Participants, consisting of 4796 men and women ages 45 to 79, were recruited between February 2004 and May 2006 at four clinical sites (University of Maryland and Johns Hopkins University, Memorial Hospital of Rhode Island, The Ohio State University, and the University of Pittsburgh). Informed consent was obtained from each participant. Radiographic images, patient-reported outcomes, and MR images were collected annually from the OAI's 12-month to 48-month visits.

For these analyses, we used data from an ongoing project that aimed to characterize longitudinal changes in the disease activity metric. The sample for the ongoing cohort study comprised individuals with at least one knee with a Kellgren-Lawrence grade≥1 and a WOMAC pain score≥10/100 at the 12-month visit to resemble a clinical trial among participants with, or at high risk of, radiographic OA and with sufficient symptoms to observe improvement or worsening knee pain. We excluded participants who underwent knee replacement during the 3-year observation period. We required data for patient-reported outcomes and walking speed at least once during the observation period (Supplemental Fig. 1). Additionally, we required participants to have bilateral MR images available from at least 3 of 4 consecutive OAI visits.

### 2.3 Study sample for current analyses

For the current analyses, we included people without sxKOA (defined below) at the 12-month OAI visit. We excluded 197 knees without 12-month disease activity measurements and seven knees because of missing outcome data. Our final study sample included 913 knees (576 people).

We also conducted exploratory analyses stratified among the three phenotypes that could develop incident symptomatic knee OA: 1) knees without symptoms nor radiographic OA (n = 277), 2) knees with radiographic OA only (no symptoms; n = 459), and 3) knees with symptoms (without radiographic OA; n = 175).

### 2.4 Knee radiographs

Bilateral weight-bearing, fixed-flexion posterior-anterior knee radiographs were acquired annually. Central readers provided Kellgren-Lawrence grades (0–4) [19]. The Kellgren-Lawrence grades had good agreement between two readings separated by 3–9 months (kappa coefficients between 0.70 and 0.78; n = 150). We relied on readings from project 15 in the OAI public files: kxr_sq_bu## (versions 1.8, 3,7, 5.7, and 6.5).

### 2.5 Knee pain evaluation

The OAI administered knee-specific WOMAC pain questionnaires, a validated score for evaluation of symptoms for knee OA [20]. Participants used these questionnaires to identify their knee pain severity on a scale of 0–20. We converted this into a scale of 0–100, with 100 being the most pain. For these analyses, we only used 12-month visit scores to determine participant eligibility.

Frequent knee pain was assessed with a standardized question (answer: yes or no): “During the last 12 months, have you had pain, aching, or stiffness in or around your right knee on most days for at least one month? By most days, we mean more than half the days of a month.” The same question was asked for the left knee. We relied on data from the OAI public files: allclinical## (versions 1.2.2., 3.2.1, 5.2.1, and 6.2.1).

### 2.6 MR imaging acquisition

The MR images were collected at each OAI site using identical Siemens 3.0 T Trio MR systems and knee coils. Study-certified, licensed MR technicians regularly conducted quality control measurements to maintain quality standards and consistency among MR equipment.

Acquisitions included a sagittal intermediate-weighted fat-suppressed (IWFS) sequence (field of view = 160 mm, slice thickness = 3 mm, skip = 0 mm, flip angle = 180°, echo time = 30 ms, recovery time = 3200 ms, 313 × 448 matrix, x-resolution = 0.357 mm, y-resolution = 0.511 mm), which was used to measure BML and effusion-synovitis volumes.

### 2.7 Measuring BMLs

We used our validated semi-automated software to measure BML volumes using an IWFS MR sequence in six regions: the medial and lateral patella, tibia, and femur [21]. One reader focused on patellar BML measurements, while another reader focused on tibiofemoral measurements. Each reader manually identified BMLs and adjusted the threshold to modify the computer-generated BML segmentation. An experienced third reader completed weekly quality assurance meetings to make necessary adjustments for consistency among measurements. Each reader had excellent intra-reader reliability (intra-class correlation coefficients [3,1 model] >0.95; n = 20). Our BML measures consisted of 6 regional BML volumes, representing the sum of the BML volume within each anatomic region (medial and lateral: patella, distal femur, proximal tibia).

### 2.8 Measuring effusion-synovitis

One reader measured effusion-synovitis using our previously validated semi-automated software on the IWFS MR sequence [18]. The reader used the software to manually adjust thresholds to segment regions of high-signal intensity and remove irrelevant areas of high-signal intensity (e.g., blood vessels, BMLs). The effusion-synovitis volume measures a combined construct of both the volume of joint fluid within the knee and inflammatory tissue involving the synovium throughout the entire knee. The reader for this measurement met weekly with another experienced effusion-synovitis reader to ensure quality and consistency among measurements. The reader had excellent intra-reader reliability (intra-class correlation coefficient [3,1 model] = 0.97; n = 20).

### 2.9 The disease activity metric

We calculated disease activity using a validated algorithm, providing a composite measure of 6 regional BML volumes and effusion-synovitis volume [18]. First, we adjusted the volumetric measures based on each participant's bone width, thereby accounting for variations in knee size. We then standardized each measure by subtracting the mean of each volumetric measure from a reference sample and dividing it by the standard deviation of that sample. The reference sample included 197 knees; 53% with moderate-severe radiographic knee OA, mean [SD] WOMAC pain score was 5.0 [3.6]. The purpose of the reference sample was not to serve as a comparator group but to provide stable scaling parameters (mean and standard deviation) for standardizing the BML and effusion-synovitis volumes to the same scale. Finally, we summed the standardized measures of effusion-synovitis and the BML volumes for the six regions (medial and lateral: distal femur, proximal tibia, and patella) to derive the disease activity metric, where higher values indicate worse disease.

### 2.10 Participant characteristics

Participants reported their gender (male, female, refused), date of birth (to calculate age at the 12-month visit), and race (open-ended question). The study staff measured a participant's weight and height to calculate their body mass index (kg/m^2^) during the 12-month visit. Physical activity was assessed with the Physical Activity Scale for the Elderly. We recorded a history of knee injury as an affirmative response to a question at baseline or 12-month follow-up. At baseline participants were asked “Have you ever injured your right knee badly enough to limit your ability to walk for at least two days?” At follow-up they were asked “Since your last annual visit to the OAI clinic about 12 months ago, have you injured your right knee badly enough to limit your ability to walk for at least two days?” Similar questions were asked for the left knee. We relied on data from the OAI public files: enrollees (version 25) and allclinical## (versions 1.2.2., 3.2.1, 5.2.1, and 6.2.1). Femorotibial angle at the 12-month visit was measured on weight-bearing, fixed-flexion posteroanterior knee radiograph [22], and the protocol and data are publicly available on the OAI website (file: kXR_FTA_Duryea01, version 1.2).

### 2.11 Statistical analysis

We used a modified Poisson model to assess the association between the disease activity metric at a single time point and incident sxKOA, adjusting for gender, age, and body mass index. The model used robust standard errors and accounted for clustering effect of the participant to adjust for the correlations between knees within each person. Incident sxKOA was the outcome. We evaluated the exposure of 12-month disease activity metric as a continuous measure (relative risk [RR] per 1 unit of measure) and tertiles (reference = lowest tertile). The models were adjusted for gender, age, and body mass index from the 12-month OAI visit. We performed a sensitivity analysis, limiting the sample to one knee per person (right knee). We also completed an additional analysis adjusting for gender, age, body mass index, physical activity, history of knee injury, and femorotibial angle.

For exploratory analyses, we stratified these analyses among the three phenotypes that could develop incident symptomatic knee OA: 1) knees without symptoms nor radiographic OA, 2) knees with radiographic OA only (no symptoms), and 3) knees with symptoms (without radiographic OA).

We also explored how the main components of disease activity (effusion-synovitis volume and BML volume; tertiles) related to incident sxKOA using the modeling approach described above. We also performed a hierarchical multiple informants model to compare the strength of the estimated association with incident sxKOA (outcome) and the disease activity metric, BML volume, and effusion-synovitis volume (high vs. low tertile). We adjusted the model for gender, age, and body mass index and accounted for clustering effect of the participant.

Finally, we created a box plot of the disease activity metric among knees with each Kellgren-Lawrence grade. The plot includes a visual reference (line) to the threshold for the highest tertile of disease activity. All analyses were performed in SAS Enterprise Guide (version 8.3; Cary, NC).

## 3 Results

Supplemental Fig. 1 depicts the selection process for this study's final group of participants. Our parent study included 1389 knees. We then selected knees without sxKOA at 12 months (n = 920 knees). We excluded 7 knees with missing data that prevented us from determining the outcome. The sample included 913 knees without sxKOA at the beginning of the observation period (n = 572 participants).

Table 1 provides the descriptive characteristics of the subcohort and OAI participants who may have been eligible for the outcome but were excluded from the analyses. The subcohort was, on average, in their early 60s, overweight, and mostly female and white. In general, the participants’ characteristics were similar between those included and excluded from the subcohort. The eligible cohort tended to include more knees with frequent symptoms (19% vs. 12%) and fewer knees with Kellgren-Lawrence grade = 0 (18% vs. 49%).

**Table 1:** Descriptive characteristics of eligible participants

	Eligible sample	Excluded Participants without Symptomatic Osteoarthritis
**Person-based characteristics**	**n = 572 participants**	**n = 3457 participants**
Age (years)	61.4 (9.0)	62.3 (9.2)
Body mass index (kg/m^2^)	29.4 (4.5)	28.1 (4.8)
Physical activity scale for the elderly	158 (83)	165 (82)
Females	322 (56%)	1996 (58%)
White^a^	470 (82%)	2822 (82%)
Black or African American^a^	93 (16%)	545 (16%)
**Knee-based characteristics**	**n = 913 knees**	**n = 6325 knees**
Kellgren-Lawrence grade		
0	168 (18%)	2945 (49%)
1	286 (31%)	1159 (19%)
2	316 (35%)	1183 (20%)
3	120 (13%)	574 (10%)
4	23 (3%)	95 (2%)
Frequent knee symptoms	175 (19%)	774 (12%)
History of knee injury^b^	275 (31%)	1548 (25%)
Femorotibial angle (degrees)^c^	−5.3 (2.4)	−5.4 (2.3)

a other races not reported due to small sample sizes

b history of knee injury missing for 12 eligible knees and 68 excluded knees without symptomatic osteoarthritis.

c femorotibial angle missing for 157 eligible knees and 2859 excluded knees without symptomatic osteoarthritis.

Table 2 shows the association between the disease activity metric (evaluated continuously and divided into tertiles) and incident sxKOA. Greater levels of the disease activity metric among knees without sxKOA were associated with a greater risk of subsequent development of incident sxKOA when evaluating the disease activity as a continuous measure and in tertiles (Table 2). For each unit greater of the disease activity metric (continuous measure), the adjusted relative risk for incident sxKOA increased by 8%. Compared to the lowest tertile, knees in the moderate and high disease activity tertiles had almost 2 to 3 times greater risk of incident sxKOA, respectively. These findings were similar when the sample was limited to one knee per person (right knee; Supplemental Table 2). Furthermore, the findings were similar but attenuated when we additionally adjusted for physical activity, history of knee injury, and femorotibial angle; specifically, for each unit greater of the disease activity metric (continuous measure), the adjusted relative risk for incident sxKOA increased by 6% (Supplemental Table 3).

**Table 2:** Worse disease activity is associated with a greater chance of incident symptomatic or radiographic OA

**Overall Study Sample**	Incident Symptomatic OA		Unadjusted Relative Risk (RR)	Adjusted RR^a^
	Absent	Present		
	n = 630	n = 283	(95% CI)	(95% CI)
Disease activity (mean (SD))	−1.37 (2.68)	−0.30 (2.66)	**1.07 (1.03**–**1.11)**^b^	**1.08 (1.04**–**1.11)**^b^
Disease activity –Tertiles low (−3.83 to −2.46)	253 (40%)	48 (17%)	REFERENCE	
Moderate (−2.44 to −1.05)	207 (33%)	99 (35%)	**2.05 (1.50–2.80)**	**1.99 (1.46–2.71)**
High (−1.04 to 24.79)	170 (27%)	136 (48%)	**2.89 (2.14–3.91)**	**2.79 (2.06–3.78)**

**No symptoms, No radiographic OA**	Incident symptomatic OA			

(n = 277)	Absent	Present	Unadjusted RR∗	

	n = 259	n = 18	(95% CI)	

Disease activity (continuous)	−1.94 (1.96)	−1.77 (1.29)	1.05 (0.93–1.19)	
Disease activity –Tertiles low (−3.83 to −2.46)	128 (49%)	7 (39%)	REFERENCE	
Moderate (−2.44 to −1.05)	84 (32%)	7 (39%)	Not calculated	
High (−1.04 to 24.79)	47 (18%)	4 (22%)	Not calculated	

**Radiographic OA only (No symptoms)**	Incident symptomatic OA			

(n = 459)	Absent	Present	Unadjusted RR∗	

	n = 211	n = 248	(95% CI)	

Disease activity (continuous)	−0.62 (3.43)	−0.22 (2.71)	1.02 (0.99–1.05)	
Disease activity –Tertiles low (−3.83 to −2.46)	58 (27%)	38 (15%)	REFERENCE	
Moderate (−2.44 to −1.05)	70 (33%)	87 (35%)	**1.42 (1.07–1.89)**	
High (−1.04 to 24.79)	83 (39%)	123 (50%)	**1.56 (1.19–2.05)**	

**Symptoms only (No radiographic OA)**	Incident symptomatic OA			

(n = 175)	Absent	Present	Unadjusted RR∗	

	n = 158	n = 17	(95% CI)	

Disease activity (continuous)	−1.43 (2.34)	0.03 (2.64)	**1.16 (1.05–1.29)**	
Disease activity –Tertiles low (−3.83 to −2.46)	66 (42%)	3 (18%)	REFERENCE	
Moderate (−2.44 to −1.05)	53 (34%)	5 (29%)	Not calculated	
High (−1.04 to 24.79)	39 (25%)	9 (53%)	Not calculated	

Notes. (a). Relative risks adjusted for age, gender, and body mass index. (b). Relative risks for disease activity as a continuous measurement are per 1 unit. Bold = statistically significant associations. ∗ Only unadjusted relative risks are reported because of the limited sample size within each stratum. Two knees could not be classified into a phenotype because the frequent knee pain status was reported as “Don't know/Unknown/Uncertain” by the participants.

To understand which phenotypes contributed to the association between the disease activity metric and the development of sxKOA, we performed an exploratory analysis, stratifying the analyses into three phenotypes based on 12-month visit status (Table 2). Of the 283 incident sxKOA cases, most (88%) originated from knees that had radiographic OA and no symptoms. Among knees with radiographic OA and no symptoms, the disease activity metric as a continuous measure was null; however, moderate-to-high (tertiles) disease activity metric was associated with approximately a 50% greater risk of incident sxKOA than those with lower disease activity. Additionally, knees that initially presented with only symptoms (no radiographic OA) contributed only 17 (6%) of the 283 cases but had the greatest estimated risk (16%) for each unit of greater disease activity metric (continuous).

When we explored the components of disease activity, we found that effusion-synovitis and BML volumes were both prognostic of future incident sxKOA. The relative risks were larger for the disease activity metric than for the individual components alone (Supplemental Table 1); however, the disease activity metric did not have a statistically significant stronger association with sxKOA than either individual component in the hierarchical multiple informants model.

**Fig. 1:**
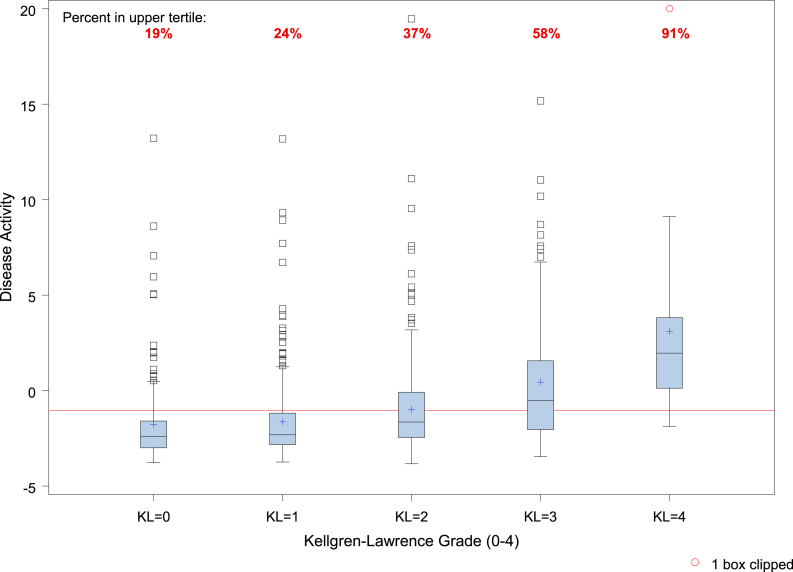
Boxplot of 12-month disease activity by 12-month Kellgren-Lawrence Grade. The red reference line is disease activity at −1.04 (disease activity above this line defined as “high disease activity” (top tertile)). 2 boxes clipped (marked by red circles) = high disease activity values beyond the upper range of the y-axis. The box represents the interquartile range (bottom = 25th percentile, top = 75th percentile, dissecting line = median). The plus sign equals the mean and the whiskers represent 1.5 times the interquartile range on either side of the 25th or 75th percentile. (For interpretation of the references to color in this figure legend, the reader is referred to the Web version of this article.)

The box plot in Fig. 1 represents the distribution of the disease activity metric among the different Kellgren-Lawrence grades (0–4). Knees with greater Kellgren-Lawrence grades were more likely to be in the upper tertile of disease activity (shown above the red threshold line).

## 4 Discussion

Our results indicate that inflammation and aberrant bone turnover, detected by MR imaging and measured using the disease activity metric, precede and are prognostic of the onset of sxKOA. Hence, these pathological processes begin earlier in the natural history of OA before a knee meets standard definitions of OA. The prognostic value of the disease activity metric may be evident in knees with or without radiographic OA (e.g., symptoms only). Our results offer evidence that the disease activity metric is a continuous metric reflecting whole-knee pathological processes before the onset of sxKOA. Measuring effusion-synovitis and BMLs using approaches such as the disease activity metric offers the potential to identify knees at an early stage, where the prospect of successful intervention may be greater, and provides a potential strategy to define or classify early knee OA.

Shifting our field's focus to people and knees with earlier OA could create an opportunity to identify disease-modifying OA therapies. Thus, there has recently been a growing focus on early intervention. The therapeutic opportunity is plausibly greatest if treatments occur at the earliest possible stage of OA – before the onset of symptomatic knee OA [[4], [5], [6], [7], [8], [9], [10], [11]]. In this context, radiographic images alone are insufficient to detect early OA. Our findings support a previous study that found radiographs to be an insensitive measurement for OA [23]. Indeed, in prior studies among knees without radiographic knee OA, effusion-synovitis and BMLs have been separately associated with incident persistent knee symptoms and incident knee OA [13,24]. Furthermore, BMLs and effusion-synovitis were linked to incident radiographic knee OA two years before its onset. We also previously demonstrated that the disease activity metric was associated with the onset of accelerated knee OA during the subsequent year [25]. In this study, we expanded these findings by using our validated composite metric to examine whether this biomarker was predictive of incident symptomatic knee OA over 3 years. We found that in knees without radiographic OA, those with high disease activity may be more likely to develop sxKOA in the subsequent 3 years. These findings support a conceptual model for the earliest phases of knee OA that emphasizes the important role of these dynamic disease processes.

Our new metrics have the potential to help define or classify knees with early OA. Many investigators have been exploring criteria for early OA that could be a screening tool to enrich study populations for clinical trials focused on intervening early in the natural history of OA [[26], [27], [28], [29]]. Disease activity is a promising candidate prognostic biomarker for screening in clinical trials, as it can be easily deployed using a 5-min MR imaging scan and measured by trained research assistants. Even within this cohort enriched for individuals at risk of knee OA, there was substantial variation in risk by disease activity level. For example, knees in the highest tertile of disease activity had nearly three times the risk of developing sxKOA compared with those in the lowest tertile. Similarly, knees with the greatest BML volume and effusion-synovitis volume had more than two-fold greater risk of incident sxKOA. These findings suggest that MRI-detected disease processes may help identify a subgroup of at-risk individuals with substantially elevated likelihood of progressing to sxKOA.

In our study, we looked at the individual metrics of BMLs and effusion-synovitis, and each was prognostic of incident sxKOA. An important benefit of the composite disease activity metric is that it combines these two dynamic features of OA, enabling the inclusion of multiple metrics in a single analysis. The combined metric allows for greater point estimates of RR for incident symptomatic knee OA, which translates to greater power of the combined metric compared with each individual feature.

Our study has some limitations. This study relied on a subcohort of a nested cohort within the OAI. While the selection criteria may have biased the sample to include more knees with frequent knee pain and Kellgren-Lawrence grades 1 or 2, it is unlikely to change the overall take-home message that dynamic disease processes antedate the onset of OA and are clinically meaningful. Furthermore, few knees without symptomatic knee OA undergo replacement within three years, excluding them may have led us to underestimate the association with incident symptomatic knee OA because greater disease activity is associated with future knee replacements [11]. Secondly, there are portions of our exploratory analyses where the cell numbers were too small to calculate relative risks, and the results should be replicated in studies enriched with these early OA phenotypes. Thirdly, the hierarchical multiple informants model may have been underpowered to detect differences in relative risks and may warrant further examination in future studies. This is also one of the first studies to look at disease activity as a prognostic biomarker for incident sxKOA. Thus, it would be helpful for these findings to be replicated before we advocate for their deployment as tools in clinical trials.

In conclusion, greater MR-based dynamic disease processes (altered bone turnover and inflammation), detected by MR imaging and measured by the disease activity metric, occur before and are prognostic of the onset of sxKOA. We offer a conceptual framework for early OA, highlighting that the disease processes are pertinent in the early stages of knee OA. Measuring effusion-synovitis and BMLs using methods such as the disease activity metric may identify knees at an early stage of OA, where the prospect of successful intervention may be greater, and provides a potential strategy for defining or classifying early knee OA. These findings can help inform the use of this composite metric as a valid measure reflecting whole-knee disease processes that can serve as a prognostic biomarker.

## Author contributions

All authors declare meeting the authorship criteria as outlined by the ICMJE. Each author has made significant contributions to the work. Jeffrey B. Driban contributed to the conception of work, data acquisition, analysis, and interpretation of data. Timothy E. McAlindon and Jonggyu Baek also contributed to the conception of work and interpretation of data. The acquisition of data was performed by Julieann C. Patarini, Emily Kirillov, Nhung Vo, Ming Zhang, and Matthew S. Harkey. Analyses and interpretation of data were provided by Jonggyu Baek, Shao-Hsien Liu, and Kate Lapane. Additional interpretation of data was offered by Matthew S. Harkey, Grace H. Lo, Charles B. Eaton, and James MacKay. All authors have approved the final version of the manuscript and agree to be accountable for all aspects of the work.

## Role of the funding source

These analyses were financially supported by a grant from the National Institute of Arthritis and Musculoskeletal and Skin Diseases of the National Institutes of Health under Award Number R01-AR076411. Dr. Harkey received support from a training grant from the National Institute of Arthritis and Musculoskeletal and Skin Diseases of the National Institutes of Health under Award Number K01-AR081389. This study was also supported by a grant R24-AR085006 from the National Institute for Arthritis and Musculoskeletal Disorders and Skin Diseases (NIAMS), National Institutes of Health. The OAI is a public-private partnership comprised of five contracts (N01-AR-2-2258; N01-AR-2-2259; N01-AR-2-2260; N01-AR-2-2261; N01-AR-2-2262) funded by the 10.13039/100000002National Institutes of Health, a branch of the Department of Health and Human Services, and conducted by the OAI Study Investigators. Private funding partners include Merck Research Laboratories; 10.13039/100008272Novartis Pharmaceuticals Corporation; 10.13039/100004330GlaxoSmithKline; and 10.13039/100004319Pfizer. Private sector funding for the OAI is managed by the 10.13039/100000009Foundation for the National Institutes of Health. This manuscript was prepared using an OAI public use data set and does not necessarily reflect the opinions or views of the OAI investigators, the NIH, or the private funding partners. This work was supported in part with resources at the VA’s Health Services Research and Development Service Center for Innovations in Quality, Effectiveness, and Safety (#CIN 13-413) at the Michael E. DeBakey VA Medical Center, Houston, TX. The views expressed in this article are those of the authors and do not necessarily represent the views of the Department of Veterans Affairs. The funding sources had no role in study design; in the collection, analysis, and interpretation of data; in the writing of the report; nor in the decision to submit the article for publication.

## Conflicts of interest

Jeffrey B. Driban declares being on the Journal of Rheumatology Editorial Board. Timothy E. McAlindon declares he is a consultant for Sanofi, Kolon TissueGene, Organogenesis, Merck, Scarcell, Genescence, BioSplice, and is the owner of Ambulomics and Arthometrics. Jeffrey B. Driban and Timothy E. McAlindon hold a patent for Objective Assessment of Joint Damage, US-20220202356, 2020. Matthew S. Harkey declares being a member of OARSI Board. All authors received funding from NIH/NIAMS R01 AR076411.

## Appendix A Supplementary data

Supplemental Fig. 1 Flow diagram demonstrating this study's participant selection process.

## References

[1] Hunter D.J., March L., Chew M. (2020). Osteoarthritis in 2020 and beyond: a lancet commission. Lancet.

[2] Lane N.E., Brandt K., Hawker G., Peeva E., Schreyer E., Tsuji W. (2011). OARSI-FDA initiative: defining the disease state of osteoarthritis. Osteoarthr. Cartil..

[3] FDA Document (August 2018).

[4] Chu C.R., Williams A.A., Coyle C.H., Bowers M.E. (2012). Early diagnosis to enable early treatment of pre-osteoarthritis. Arthritis Res. Ther..

[5] Luyten F.P., Denti M., Filardo G., Kon E., Engebretsen L. (2012). Definition and classification of early osteoarthritis of the knee. Knee Surg. Sports Traumatol. Arthrosc..

[6] Felson D.T., Hodgson R. (2014). Identifying and treating preclinical and early osteoarthritis. Rheum. Dis. Clin. N. Am..

[7] Migliore A., Scirè C.A., Carmona L., Herrero-Beaumont G., Bizzi E., Branco J. (2017). The challenge of the definition of early symptomatic knee osteoarthritis: a proposal of criteria and red flags from an international initiative promoted by the Italian society for rheumatology. Rheumatol. Int..

[8] Katz J.N., Neogi T., Callahan L.F., Block J.A., Conaghan P.G., Simon L.S. (2020). Disease modification in osteoarthritis; pathways to drug approval. Osteoarthr Cartil Open.

[9] Hawker G.A., Lohmander L.S. (2021). What an earlier recognition of osteoarthritis can do for OA prevention. Osteoarthr. Cartil..

[10] Driban J.B., Harkey M.S., McAlindon T.E., Lo G.H. (2023). The importance of context and intent when defining early-stage knee osteoarthritis. Osteoarthr. Cartil..

[11] Driban J., Harkey M., Price L., Lo G., Pang J., Zhang M. (2019). A novel composite Score reflecting disease activity predicts future knee replacements: data from the Osteoarthritis initiative [abstract]. Arthritis Rheumatol..

[12] Driban J.B., Price L., Lo G.H., Pang J., Hunter D.J., Miller E. (2013). Evaluation of bone marrow lesion volume as a knee osteoarthritis biomarker--longitudinal relationships with pain and structural changes: data from the osteoarthritis initiative. Arthritis Res. Ther..

[13] Roemer F.W., Kwoh C.K., Hannon M.J., Hunter D.J., Eckstein F., Fujii T. (2015). What comes first? Multitissue involvement leading to radiographic osteoarthritis: magnetic resonance imaging-based trajectory analysis over four years in the osteoarthritis initiative. Arthritis Rheumatol..

[14] Collins J.E., Losina E., Nevitt M.C., Roemer F.W., Guermazi A., Lynch J.A. (2016). Semiquantitative imaging biomarkers of knee Osteoarthritis progression: data from the foundation for the national Institutes of Health Osteoarthritis biomarkers consortium. Arthritis Rheumatol..

[15] Roemer F.W., Guermazi A., Collins J.E., Losina E., Nevitt M.C., Lynch J.A. (2016). Semi-quantitative MRI biomarkers of knee osteoarthritis progression in the FNIH biomarkers consortium cohort - methodologic aspects and definition of change. BMC Muscoskelet. Disord..

[16] Sharma L., Hochberg M., Nevitt M., Guermazi A., Roemer F., Crema M.D. (2017). Knee tissue lesions and prediction of incident knee osteoarthritis over 7 years in a cohort of persons at higher risk. Osteoarthr. Cartil..

[17] Zhang Y., Nevitt M., Niu J., Lewis C., Torner J., Guermazi A. (2011). Fluctuation of knee pain and changes in bone marrow lesions, effusions, and synovitis on magnetic resonance imaging. Arthritis Rheum..

[18] Driban J.B., Price L.L., LaValley M.P., Lo G.H., Zhang M., Harkey M.S. (2022). Novel framework for measuring whole knee osteoarthritis progression using magnetic resonance imaging. Arthritis Care Res..

[19] Kellgren J.H., Lawrence J.S. (1963).

[20] Bellamy N., Buchanan W.W., Goldsmith C.H., Campbell J., Stitt L.W. (1988). Validation study of WOMAC: a health status instrument for measuring clinically important patient relevant outcomes to antirheumatic drug therapy in patients with osteoarthritis of the hip or knee. J. Rheumatol..

[21] Zhang M., Driban J.B., Price L.L., Lo G.H., McAlindon T.E. (2015). Magnetic resonance image sequence influences the relationship between bone marrow lesions volume and pain: data from the Osteoarthritis initiative. Biomed Res. Int..

[22] Iranpour-Boroujeni T., Li J., Lynch J.A., Nevitt M., Duryea J., Investigators O.A.I. (2014). A new method to measure anatomic knee alignment for large studies of OA: data from the osteoarthritis initiative. Osteoarthr. Cartil..

[23] Roemer F.W., Kwoh C.K., Fujii T., Hannon M.J., Boudreau R.M., Hunter D.J. (2018). From early radiographic knee osteoarthritis to joint arthroplasty: determinants of structural progression and symptoms. Arthritis Care Res..

[24] Sharma L., Chmiel J.S., Almagor O., Dunlop D., Guermazi A., Bathon J.M. (2014). Significance of preradiographic magnetic resonance imaging lesions in persons at increased risk of knee osteoarthritis. Arthritis Rheumatol..

[25] Harkey M.S., Davis J.E., Price L.L., Ward R.J., MacKay J.W., Eaton C.B. (2020). Composite quantitative knee structure metrics predict the development of accelerated knee osteoarthritis: data from the osteoarthritis initiative. BMC Muscoskelet. Disord..

[26] Luyten F.P., Bierma-Zeinstra S., Dell'Accio F., Kraus V.B., Nakata K., Sekiya I. (2018). Toward classification criteria for early osteoarthritis of the knee. Semin. Arthritis Rheum..

[27] Hunter D.J., Arden N., Conaghan P.G., Eckstein F., Gold G., Grainger A. (2011). Definition of osteoarthritis on MRI: results of a Delphi exercise. Osteoarthr. Cartil..

[28] Harkey M.S., Baez S., Lewis J., Grindstaff T.L., Hart J., Driban J.B. (2021). Prevalence of early knee Osteoarthritis illness among various patient-reported classification criteria after anterior cruciate ligament reconstruction. Arthritis Care Res..

[29] Liew J.W., King L.K., Mahmoudian A., Wang Q., Atkinson H.F., Flynn D.B. (2023). A scoping review of how early-stage knee osteoarthritis has been defined. Osteoarthr. Cartil..

