# Supplementary material for: Early detection of knee osteoarthritis – The role of a composite disease activity metric: Data from the Osteoarthritis Initiative
Source: Osteoarthr Cartil Open. 2026 May 5;8(2):100811. doi: 10.1016/j.ocarto.2026.100811 (PMC13196552; doi:10.1016/j.ocarto.2026.100811)

| **Supplemental Table 1. Worse Disease Activity is Associated with a Greater Chance of Developing Incident Symptomatic or Radiographic Osteoarthritis (OA)** | | | | |
| --- | --- | --- | --- | --- |
|  | Incident Symptomatic OA | |  |  |
|  | Absent | Present | Unadjusted  Relative Risk | Adjusted  Relative Risk^a^ |
|  | n = 630 | n = 283 | (95% CI) | (95% CI) |
| Effusion-Synovitis - Tertiles |  |  |  |  |
| Low (2.23 to 7.68) | 238 (38%) | 65 (23%) | REFERENCE |  |
| Moderate (7.70 to 11.40) | 219 (35%) | 85 (30%) | 1.28 (0.97 - 1.69) | **1.35 (1.03 - 1.77)** |
| High (11.41 to 81.35) | 173 (27%) | 133 (47%) | **2.09 (1.61 - 2.70)** | **2.20 (1.71 - 2.84)** |
| BML Volume - Tertiles |  |  |  |  |
| Low (0.00 to 0.57) | 249 (40%) | 54 (19%) | REFERENCE |  |
| Moderate (0.57 to 2.81) | 213 (34%) | 91 (32%) | **1.77 (1.31 - 2.38)** | **1.77 (1.31 - 2.38)** |
| High (2.83 to 87.82) | 168 (27%) | 138 (49%) | **2.71 (2.05 - 3.58)** | **2.64 (2.00 - 3.49)** |
| Notes.  a. Relative risks adjusted for age, gender, and body mass index.  b. Relative risks for disease activity as a continuous measurement is per 1 unit.  Bold = statistically significant associations | | | | |

| **Supplemental Table 2. Worse Disease Activity is Associated with a Greater Chance of Incident Symptomatic or Radiographic OA (Right Knees Only)** | | | |
| --- | --- | --- | --- |
|  | Incident Symptomatic OA | |  |
|  | Absent | Present | Adjusted RR^a^ |
| **Overall Study Sample (Right Knees Only)** | n= 312 | n = 140 | (95% CI) |
| Disease Activity (mean (SD)) | -1.41 (2.16) | -0.08 (2.88) | **1.12 (1.08 - 1.16)**^b^ |
| Disease Activity –Tertiles Low (-3.83 to -2.46) | 121 (39%) | 21 (15%) | REFERENCE |
| Moderate (-2.44 to -1.05) | 100 (32%) | 52 (37%) | **2.14 (1.37 – 3.35)** |
| High (-1.04 to 24.79) | 91 (29%) | 67 (48%) | **2.69 (1.73 – 4.17)** |
| Notes. (a). Relative risks adjusted for age, gender, and body mass index. (b). Relative risks for disease activity as a continuous measurement are per 1 unit. Bold = statistically significant associations. | | | |

| **Supplemental Table 3. Worse Disease Activity is Associated with a Greater Chance of Incident Symptomatic or Radiographic OA (Additional Adjustments)**^a^ | | | |
| --- | --- | --- | --- |
|  | Incident Symptomatic OA | |  |
|  | Absent | Present | Adjusted RR^a^ |
| **Overall Study Sample (Right Knees Only)** | n= 465 | n = 275 | (95% CI) |
| Disease Activity (mean (SD)) | -1.21 (2.80) | -0.29 (2.68) | **1.06 (1.03 - 1.10)** |
| Disease Activity –Tertiles Low (-3.83 to -2.46) | 172 (37%) | 47 (17%) | REFERENCE |
| Moderate (-2.44 to -1.05) | 156 (34%) | 97 (35%) | **1.83 (1.35 - 2.47)** |
| High (-1.04 to 24.79) | 137 (29%) | 131 (48%) | **2.39 (1.78 - 3.21)** |
| Notes. (a). Relative risks adjusted for age, gender, body mass index, physical activity (Physical Activity Scale for the Elderly), history of knee injury, and femorotibial angle. (b). Relative risks for disease activity as a continuous measurement are per 1 unit. Bold = statistically significant associations. | | | |

**Supplemental Figure 1.**


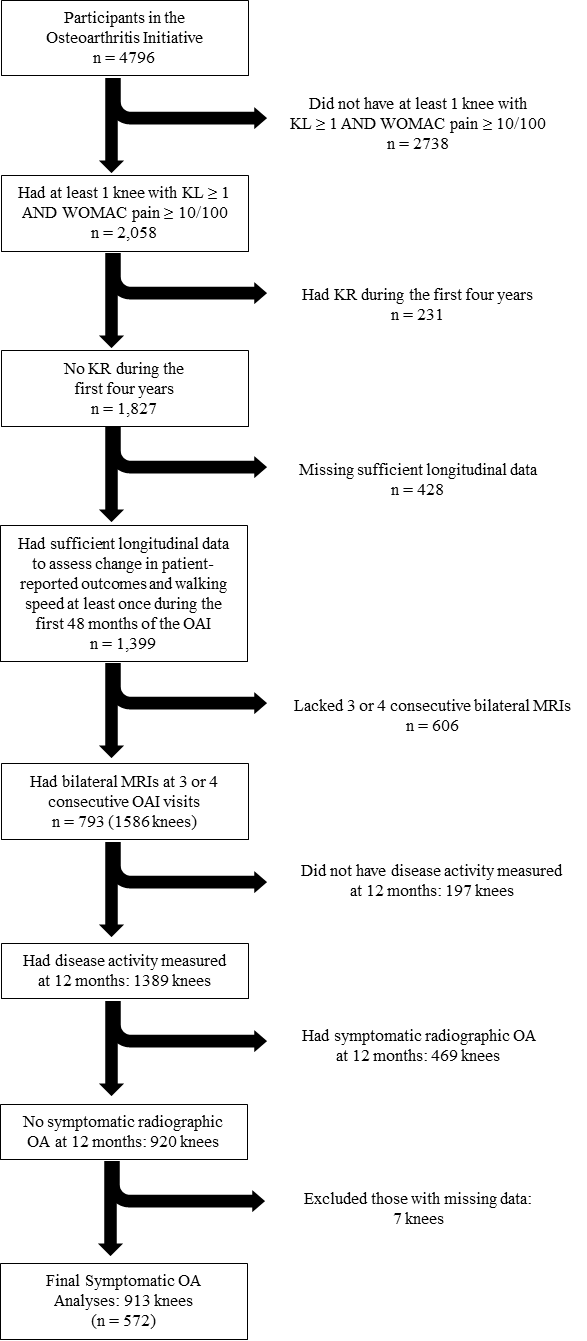

Supplement: Supplemental Fig. 1 — Flow diagram demonstrating this study's participant selection process. [file mmc1.docx]
